# Metabolic Landscape of a Genetically Engineered Mouse Model of IDH1 Mutant Glioma

**DOI:** 10.3390/cancers12061633

**Published:** 2020-06-19

**Authors:** Victor Ruiz-Rodado, Tomohiro Seki, Tyrone Dowdy, Adrian Lita, Meili Zhang, Sue Han, Chunzhang Yang, Murali K. Cherukuri, Mark R. Gilbert, Mioara Larion

**Affiliations:** 1 Neuro-Oncology Branch, National Cancer Institute, Center for Cancer Research, National Institute of Health, Bethesda, MD 20814, USA; victor.ruizrodado@nih.gov (V.R.-R.); tyrone.dowdy@nih.gov (T.D.); adrian.lita@nih.gov (A.L.); zhanmeil@mail.nih.gov (M.Z.); sue.han@nih.gov (S.H.); Chungzhang.Yang@nih.gov (C.Y.); mark.gilbert@nih.gov (M.R.G.); 2 Radiation Biology Branch, Center for Cancer Research, National Institute of Health, Bethesda, MD 20814, USA; tomos511@josai.ac.jp (T.S.); cherukum@mail.nih.gov (M.K.C.)

**Keywords:** genetically engineered mouse models, IDH1-mutant gliomas, ^13^C-tracing, 2-hydroxyglutarate formation

## Abstract

Understanding the metabolic reprogramming of aggressive brain tumors has potential applications for therapeutics as well as imaging biomarkers. However, little is known about the nutrient requirements of isocitrate dehydrogenase 1 (IDH1) mutant gliomas. The IDH1 mutation involves the acquisition of a neomorphic enzymatic activity which generates D-2-hydroxyglutarate from α-ketoglutarate. In order to gain insight into the metabolism of these malignant brain tumors, we conducted metabolic profiling of the orthotopic tumor and the contralateral regions for the mouse model of IDH1 mutant glioma; as well as to examine the utilization of glucose and glutamine in supplying major metabolic pathways such as glycolysis and tricarboxylic acid (TCA). We also revealed that the main substrate of 2-hydroxyglutarate is glutamine in this model, and how this re-routing impairs its utilization in the TCA. Our ^13^C tracing analysis, along with hyperpolarized magnetic resonance experiments, revealed an active glycolytic pathway similar in both regions (tumor and contralateral) of the brain. Therefore, we describe the reprogramming of the central carbon metabolism associated with the IDH1 mutation in a genetically engineered mouse model which reflects the tumor biology encountered in glioma patients.

## 1. Introduction

Isocitrate dehydrogenase 1 (IDH1) mutations are genetic alterations that are considered driver mutations and occur in 70–90% of lower-grade gliomas (LGG) [1]; however, faithful models that recapitulate this disease are sparse. Genetically engineered mouse models (GEMM) offer an alternative to represent the characteristic phenotype associated with these tumors and to investigate the metabolic reprogramming associated with the isocitrate dehydrogenase 1 mutation (IDH1*^mut^*). The GEMM employed herein involves the concomitant overexpression of IDH1*^mut^* with platelet-derived growth factor (PDGF) (*NTVa-Inka Arf^−/−^Pten* flox/flox) and showed many similarities to the human disease, including similar histological pattern, the production of 2-hydroxyglutarate (2HG), increased DNA methylation, and similar gene expression differences relative to wild type (IDH*^wt^*) mouse tumors [2]. Metabolism-related genes have been previously reported to have a differential expression in this model compared to their wild type (WT) counterpart [2]; indeed, IDH1*^wt^* and IDH1*^mut^* gliomas are biologically distinct tumors with solid differences in their molecular profiles beyond the IDH1 status [3]. IDH1 mutations are considered early events in tumorigenesis [4]; therefore, the subsequent incorporation of further mutations includes more variability between IDH1 mutant and wild-type gliomas. Dissecting the metabolic abnormalities specifically occurring in IDH1*^mut^* in contrast with the normal tissue may reveal tumor vulnerabilities that can be exploited for therapy. Tumor cells reshape their metabolism in order to sustain their rapid growth [5] and this remodeling can be further modified in those cases where mutations occur in pivotal metabolic enzymes, such as IDH1. IDH1 are nicotinamide adenine dinucleotide phosphate (NADP^+^)-dependent enzymes that catalyze the reversible reaction of isocitrate to α-ketoglutarate (αKG), yielding reduced nicotinamide adenine dinucleotide phosphate NADPH and CO_2_. The cancer-associated mutation of IDH has gained a neomorphic activity by catalyzing the conversion of αKG into 2HG while oxidizing NADPH. This reaction produces a 50- to 100-fold increase in 2HG levels in cells harboring the mutation [6,7]. This mutation is considered to be a major feature in reshaping the metabolic landscape of tumors such as gliomas [8,9] or fibrosacromas [10], along with a defective activity of the WT reaction, which reversibly interconverts isocitrate and αKG [11]. However, it is not clear whether the IDH1 mutation by itself is sufficient to describe the metabolic complexity and heterogeneity of these tumors in time and space. The carbon source of 2HG is still under investigation, although recent reports have linked glutamine (Gln) to 2HG formation [12,13], which may involve the rewiring of central carbon metabolism in view of its importance as a major substrate for this route in cancer cells [14]. Using nuclear magnetic resonance (NMR) and mass spectrometry-based ^13^C-tracing in addition to hyperpolarized magnetic resonance spectral imaging (MRSI), we provide a comprehensive metabolic characterization of this mouse model in comparison with normal tissue, which can serve as a reference metabolic landscape for IDH1*^mut^* gliomas. The findings revealed herein can be employed for selective targeting of dysregulated metabolic pathways.

## 2. Results

### 2.1. 2-Hydroxyglutarate and Amino Acid Metabolism in IDH1^mut^ Glioma

**Figure 1:**
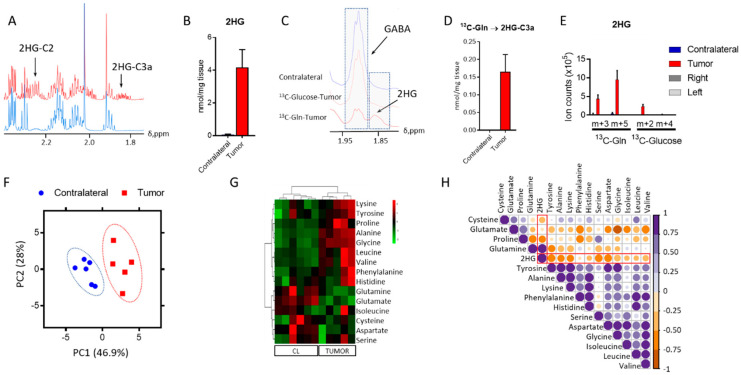
2-Hydroxyglutarate (2HG) and amino acid metabolism in isocitrate dehydrogenase 1 mutation (IDH1*^mut^*) glioma. (**A**) 2HG identification in the tumor tissue obtained from the mouse model. (**B**) Quantification of 2HG from direct integration of the resonance signal centered at 1.83 ppm. (**C**) Stack plot of 1D heteronuclear single quantum coherence (1D-HSQC) spectra from the contralateral and tumor regions from mice infused with either ^13^C-U-Glutamine (Gln) or ^13^C-U-Glucose. (**D**) Contribution of ^13^C-U-Gln to 2HG formation via nuclear magnetic resonance (NMR), together with ^13^C-U-Glucose via liquid chromatography–mass spectrometry (LC-MS). (**E**,**F**) Principal component analysis (PCA) scores plot from the metabolite levels obtained by ^1^H NMR analysis displaying the clustered samples by class (tumor and contralateral/tumor-free). (**G**) Heat-map including the amino acids identified in our analysis. CL = contralateral tissue. (**H**) Correlation map for the amino acids detected through our analysis and 2HG in the tumor tissue only. Correlation assessed through the computation of Pearson coefficient r and displayed according to the color code bar on the right. (*n* = 5–6, for all the analyses). 2HG correlations are highlighted in red.

One major question in the field of glioma with IDH1 mutations is related to the source of 2HG synthesis. While 2HG formation was previously reported in this model, the assignment of a substrate for its synthesis was not [2]. Knowing the source of 2HG could inform on the details of metabolic rewiring experienced by the cancer cells to overcome this demand. Therefore, we conducted a ^13^C tracing analysis utilizing both ^13^C-U-Glucose and ^13^C-U-Glutamine, which identified the latter as the main metabolite that contributes towards its synthesis. We firstly identified the resonance arising from this oncometabolite in an ^1^H-NMR spectrum (Figure 1A) to subsequently quantify it (Figure 1B) through direct integration of the resonance centered at 1.83 ppm which arises from the proton linked to C3 of 2HG [15]. We did not observe this resonance in the 1D heteronuclear single quantum coherence (HSQC) spectrum acquired from mice infused with ^13^C-U-Glucose; however, we detected and quantified this signal in the glutamine infused mice (Figure 1C,D). The findings observed via NMR were further confirmed by liquid chromatography–mass spectrometry (LC-MS) (Figure 1E) analysis of tumor extracts collected from this mouse model. This data shows that in this GEMM model of IDH1*^mut^* glioma, 2HG is synthesized primarily from glutamine.

To understand the overall metabolic changes that arise as a result of 2HG formation, we conducted untargeted metabolic profiling of IDH1*^mut^* tumor and normal brain. We explored the polar metabolic profile of these tumors and compared them to the tumor-free (contralateral, CL) region of the brain. Principal component analysis (PCA) (Figure 1F) scores plot revealed a clear distinction of both groups based on the concentration of 30 metabolites quantified by ^1^H NMR, excluding 2HG. Biomolecules such as amino acids, identified in the metabolic profiles of GEMM, have been recently highlighted as potential metabolic markers of central nervous system (CNS) tumors [16,17], and as metabolites dysregulated as a function of IDH1 mutation [18]. Therefore, we analyzed amino acid levels in both tumor and contralateral regions. Aspartate (Asp), glutamate (Glu), and glutamine (Gln) displayed lower levels in tumor tissue (Figure 1G) compared to those in the contralateral region, which may have arisen from the utilization of Gln as a 2HG precursor and its additional function as a precursor of Glu, and subsequently, Asp. The top upregulated amino acids in the tumor were lysine, tyrosine, proline, alanine, and glycine. Levels of brain-specific metabolites such as γ-aminobutyric acid (GABA) or N-acetylaspartate (NAA) were also computed in addition to glutathione (GSH) (Figure S1), which has been reported as a dysregulated metabolite in tumors in which IDH was knocked down [19]. NAA presented significantly different levels between the tumor and CL regions, whilst both GSH and GABA seemed unaffected; indeed, NAA is a direct product of Asp that was found to be downregulated in the tumor region when compared to the tumor-free area (Figure 1G). Moreover, we conducted a correlation analysis of the amino acids detected through our analytical platforms to unravel the connections between these metabolites and 2HG in the tumor tissue. Although we found strong correlations for some of the amino acids, only those computed for lysine and alanine (*p* < 0.05, r = 0.9) and glycine and glutamate (*p* < 0.05, r = −0.9) displayed uncorrected significant *p*-values. 2HG was positively correlated to Gln only, and we found a negative correlation to the remaining amino acids, being stronger for Asp, serine, and proline.

### 2.2. IDH1^mut^ Tumors Show Lactate Dehydrogenase A (LDHA) Activity

**Figure 2:**
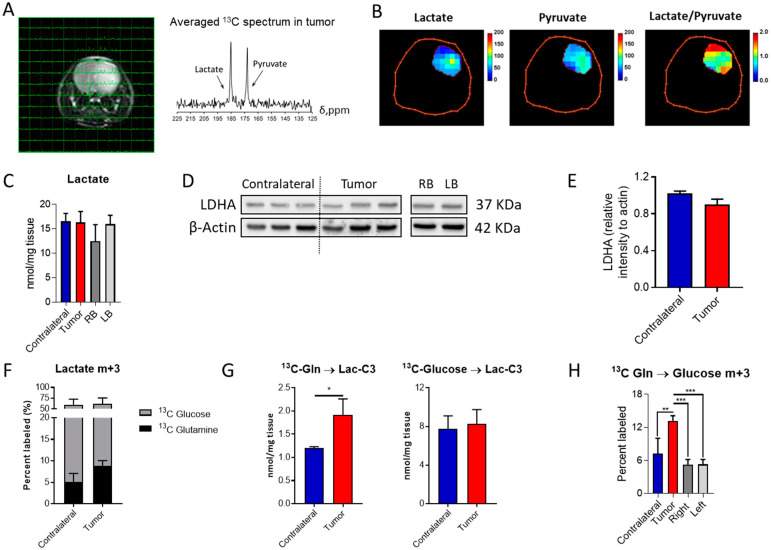
Lactate formation in aggressive IDH1*^mut^* gliomas: (**A**) MRI of the mouse brain with overlaid ^13^C NMR spectra for each voxel and the averaged spectrum for the tumor region. (**B**) Heat-maps of lactate and pyruvate levels in addition to those for the lactate/pyruvate ratio in the tumor region. (**C**) Quantification of lactate in the different regions of the brain and mice (RB and LB refer to the right- and left-brain hemispheres, respectively). (**D**) Western blot of lactate dehydrogenase A (LDHA) and its (**E**) quantification (*n* = 3). (**F**) Percent of lactate m+3 labeled and (**G**) its quantification when infused with the two ^13^C probes. (**H**) Percent of glucose m+3 labeled from ^13^C-U-Gln as a marker of gluconeogenic activity. ^13^C tracing analysis involved *n* = 5–6 mice/group and statistical significance was assessed by *t*-test for 2 group comparisons, or by ANOVA following Tukey’s multiple comparison test. *, *p* < 0.05; **, *p* < 0.005; ***, *p* < 0.001.

Lactate production under aerobic conditions, the so-called Warburg effect, is usually encountered in tumor cells, although IDH1*^mut^* gliomas have been traditionally defined as non-glycolytic [20], and thought to mainly divert pyruvate towards oxaloacetate through pyruvate carboxylase [21]. Based on our previous observations [22], these tumors can display an aggressive phenotype which is accompanied by an active glycolytic pathway yielding lactate. Hence, we explored lactate dehydrogenase (LDH) activity in this mouse model through hyperpolarized MRSI to evaluate the generation of lactate from pyruvate non-invasively (Figure 2A,B and Figure S2A,B). Glycolytic pathway activity was also assessed through ^13^C-glucose infusion and subsequent NMR and LC-MS analyses. Lactate tumor levels were similar to those encountered in the contralateral region, as well as those found in the right- and left-brain hemispheres (Figure 2C) based on tissue obtained from the control mouse model, which serves as the biological frame for tumor formation. Likewise, LDHA levels were similar in those cases investigated herein (Figure 2D,E and Figure S4). Lactate is mainly derived from glucose in both the contralateral and tumor regions (Figure 2F), and this contribution is unaffected by the disease. However, an increased flux from Gln to lactate was detected (Figure 2G) in addition to an increase in gluconeogenic activity, since higher levels of glucose m+3 isotopologue were observed within the tumor tissue collected from mice infused with ^13^C-U-Gln (Figure 2H). This glucose-derived Gln can then initiate the glycolytic route and produce m+3 lactate. Interestingly, these results suggest that these tumors are rewiring their metabolism to utilize glutamine for lactate synthesis.

### 2.3. Tricarboxylic Acid (TCA) Cycle in IDH1^mut^ Glioma

**Figure 3:**
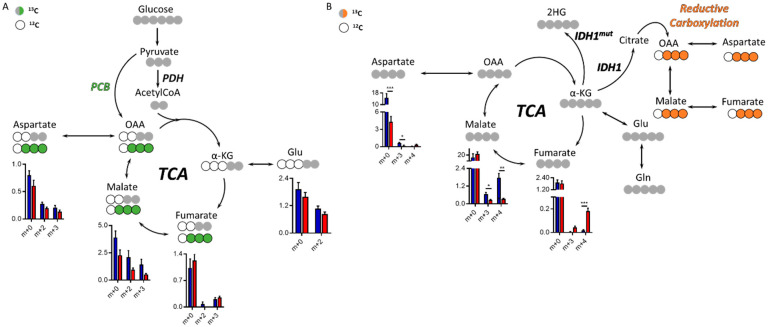
Tricarboxylic acid (TCA) cycle in the IDH1*^mut^* patient-derived indolent and genetically engineered mouse model (GEEM) glioma. (**A**) Simplified diagram of ^13^C-tracing experiment utilizing ^13^C-U-Glucose or (**B**) ^13^C-U-Glutamine. Levels of relative intensities for each isotopologue from the TCA cycle metabolite are shown for tumors (red) and contralateral regions (blue). Statistical significance for each isotopologue was assessed by multiple *t*-tests including Welch’s correction (*n* = 5–6). *, *p* < 0.05; **, *p* < 0.005; ***, *p* < 0.001. (*n* = 5–6 animals per cohort and tracer). PCB = pyruvate carboxylase, PDH = pyruvate dehydrogenase, OAA = oxaloacetate, α-KG = α-ketoglutarate.

Both glucose and glutamine are major nutrients of tumor cells, and understanding the preference for one of these substrates could inform on specific metabolic reprogramming. We therefore characterized the glucose and glutamine contribution to TCA cycle intermediate synthesis via ^13^C labeling in this model of IDH1*^mut^* glioma. There are two main entries into the TCA from glucose, one through pyruvate carboxylase (PCB)-yielding oxaloacetate (OAA) and the other through pyruvate dehydrogenase (PDH), which generates acetyl-CoA that can be subsequently combined with OAA to form citrate within the TCA. PCB activity generates m+3 isotopologues and PDH m+2 (Figure 3A), and the regulation of both enzymatic activities have been linked to 2HG [21,23]. Although we did not observe a significant change of these two enzymatic activities, malate levels were higher in the contralateral group (without attaining statistical significance, although *p* = 0.059, 0.054, and 0.053 for m+0, m+1, and m+2, respectively), which is also observed in the ^13^C-Gln tracing experiment. ^13^C-U-Gln can generate m+4 isotopologues through the oxidative TCA following glutaminolysis, or m+3 species through reductive carboxylation outside the mitochondria (Figure 3B), an activity which requires IDH1*^wt^* activity. Asp and malate m+3 levels (from ^13^C-U-Gln) were lower in the tumor area than those computed in the contralateral region. Additionally, fumarate m+4 levels in the tumor were higher and malate m+4 lower, which may indicate a defect in the flux of fumarate towards malate. Total Asp levels were also significantly lower in the tumor tissue, mainly due to the reduced flux from Gln, which is in accordance with the results obtained in the untargeted profiling for Asp and NAA (Figure 1G and Figure S1).

## 3. Discussion

Metabolic profiling of tissue can serve as a main driver for discovery of novel therapeutic strategies, since we can infer dysregulated enzymatic reactions that might be valuable clinical targets [24,25,26]. Actually, a hallmark of cancer progression is the metabolic reprogramming of tumors [27], an event which may be even more remarkable in those tumors harboring mutations in key metabolic enzymes. Therefore, we set out to provide a metabolic landscape of a mouse model of IDH1*^mut^* glioma (a fatal disease without an effective available therapy), which displays some of the characteristic features of the human malignancy, such as higher survival than its wild-type counterpart and a diffuse pattern throughout the brain (Figure S3B,C).

Metabolic profiles of tumor tissue and contralateral regions were clearly distinct with major dysregulated metabolites including both Glu and Gln. The levels of these amino acids were decreased in IDH1*^mut^* tissue, and accordingly, their metabolic products displayed the same trend, such as Asp, NAA, and GSH. These observations are in accordance with previous investigations using magnetic resonance spectroscopy (MRS) on glioma patients [28], and human tissue in which Glu and Gln were downregulated as a function of IDH1*^mut^* tumors [29,30,31]. Additionally, the levels of alanine were significantly higher in the tumor tissue, an amino acid which can be derived from pyruvate through glutamate-pyruvate transaminase (GPT) activity; alternatively, pyruvate can be reduced to lactate-yielding nicotinamide adenine dinucleotide NAD^+^, a reaction catalyzed by LDHA. The activity of LDHA assessed by hyperpolarized-MRSI utilizing ^13^C-1-pyruvate has been proposed as a biomarker of response to treatment [32] and aggressiveness in gliomas [22]. Interestingly, MRS investigations have also identified lactate resonances in both IDH1 and IDH2 brain malignancies [33], and described IDH2 gliomas as more glycolytic than those harboring the IDH1 mutation [34]. Herein, we found that LDHA expression and lactate levels in both the tumor and contralateral areas are alike; similar to the results previously reported in a patient-derived GBM tumor [35]. We observed an increase in the flux from Gln to lactate in the tumor region, accompanied by elevated levels of glucose m+3 when utilizing ^13^C-Gln as a tracer, which is an indicator of gluconeogenic activity. Therefore, the higher levels of Gln-derived lactate were not reflecting a higher Warburg-like effect in IDH1*^mut^* gliomas; rather, an increased gluconeogenic activity of this tumor, although the contribution of Gln to the total lactate pool was much lower (8.91 ± 1.01%) than that from glucose (54.69 ± 10.87%). Indeed, total lactate levels between both brain regions did not attain statistical significance despite the higher glutamine flux towards lactate. Gluconeogenic activity in glioma was previously observed in vitro when glucose was substituted by pyruvate in GBM cell lines [36], and other tumors such as lung cancer have also shown gluconeogenesis activity from Gln [37]. Additionally, gluconeogenic activity is known to be dysregulated in astrocytes [38]; however, this metabolic activity was not yet been evaluated in IDH1*^mut^* gliomas. A confounding factor could be the extra-CNS synthesis of glucose from the ^13^C-infused Gln, subsequent transportation through the bloodstream into the brain, and incorporation into the metabolism. Nevertheless, this process would involve either an enhanced extra-CNS gluconeogenic activity or an increased glucose uptake of tumor. Herein, the total glucose levels were found to be similar in tumor tissue and contralateral regions (Figure S2D).

## 4. Materials and Methods

### 4.1. Genetically Engineered Mouse Models (GEMM)

A mouse glioma model with an IDH1 R132H mutation was established based on the previously described RVAS/tva system [2,39]. Twenty-eight-day-old *Ntva_Ink4a/Arf^−/−^* mice were used for tumor modeling. Chicken fibroblast cells DF1 (ATCC, Manassas, VA, USA) were transfected with plasmids coding RCAS-PDGFa or RCAS-IDH1R132H-shp53 (Figure S3A) through Lipofectamine 2000 (Thermo Fisher, Waltham, MA, USA). Equal volumes of transfected DF-1 cells were mixed and injected in the cerebral cortex of the mice through a stereotactic procedure. All animal experiments were conducted in accordance with the principles and procedures outlined in the National Institutes of Health (NIH) Guide for the Care and Use of Animals and approved by the Animal Care and Use Committee of the NIH under the protocol NOB-008.

### 4.2. Tissue Extraction for Metabolomics Analyses

Tissue from GEMM or normal mice was weighed as frozen (~60 mg) and metabolites were extracted by tissue homogenization utilizing a bullet blender in a 1:2:2 water:methanol:chloroform solution. Then, samples were centrifuged at 12,000 rpm, for 20 min at 4 °C. The two resulting phases (upper aqueous polar and lower organic lipid) were separated and the protein interface was saved for western blotting. Hydrophobic and hydrophilic phases were split in half for NMR and LC-MS analysis and dried under a stream of N_2_. Five to six mice were utilized for each cohort (GEMM and normal mice) and ^13^C tracer.

### 4.3. NMR

For NMR, the dried sediment was reconstituted in 180 µL of a 100 mM pH 7 phosphate buffer in 100% D_2_O (0.05% wt. d-TSP) and transferred to a 3 mm-NMR tube for spectral acquisition. All the spectra were acquired at 25 °C on a Bruker AVANCE III 600 MHz spectrometer (Structural Biophysics Laboratory, NCI, Frederick, MD, USA) equipped with a cryogenically cooled probe. Single pulse ^1^H NMR experiments were performed using the noesygppr1d (TopSpin 3.5, Bruker Biospin, Billerica, MA, USA) pulse sequence. For each spectrum, 128 scans were acquired, with a relaxation delay of 3 s, a spectral width of 10,800 Hz, and a time domain of 32K points. Spectra were referenced to the TSP internal standard signal (s, δ = 0.00 ppm), zero-filled to 64K points, phased, and baseline-corrected using ACD Labs Spectrus Processor 2016 (Advanced Chemistry Development, Inc., Toronto, ON, Canada), and an exponential line broadening function of 0.30 Hz was applied. For quantification, ^1^H NMR resonance signals were integrated using ACD Labs Spectrus Processor 2016, normalized to the TSP singlet located at 0.00 ppm, and corrected to the tissue weight. 1D-HSQC (heteronuclear single quantum coherence) spectra were acquired for 768 scans, a time domain of 3.5K, a delay of 1.75 s, and a spectral width of 8 KHz. The spectral processing involved the application of exponential line broadening function of 4 Hz and a Gaussian function of 7.5 Hz. Quantification of ^13^C-labeled metabolites was performed as previously described [40]. NMR resonance signals were assigned on the basis of literature values [41,42] and information available in databases such as the Human Metabolome Database (HMDB) [43].

### 4.4. Liquid Chromatography–Mass Spectrometry (LC-MS)

Tissue hydrophilic extracts were resuspended in 60% methanol (aq) and acquired on the Agilent 6545 Qtof-MS (Agilent Technologies, Wilmington, DE, USA) with Infinity II 1290 UHPLC (Agilent Technologies, Wilmington, DE, USA). LC-MS data acquisition was conducted through polar assay developed to achieve broad detection and high resolution of amino acids, sugar phosphates, and central carbon metabolites. Global profiling, relative quantification for steady state, and time-dependent ^13^C-label flux analyses of polar metabolites was conducted on both the AdvanceBio Glycan Map 2.1 × 100 mm 2.7 µm column with Infinity II in-line filter (Agilent Technologies, Wilmington, DE, USA). Only LC-MS grade solvents and additives (Covachem, Loves Park, IL, USA) were used to prepare reagents, mobile phases and wash solutions, unless otherwise indicated. Wash cycles consisting of strong wash (50% methanol, 25% isopropanol, and 25% water), weak wash (90% acetonitrile and 10% water), and seal wash (10% isopropanol and 90% water) were utilized to eliminate carryover between consecutive injections. Glycan Map HILIC (Agilent Technologies, Wilmington, DE, USA) acquisition was performed in two experiments: Both positive and negative electrospray ionization (ESI) modes. Compounds were resolved over mobile phase A (10 mM ammonium acetate in 88% water and 12% acetonitrile, pH 6.85) and mobile phase B (10 mM ammonium acetate in 90% acetonitrile, pH 6.85) with column temperature 30 °C at flow rate 0.25 mL/min under gradient conditions: 100% B, 0.5 min; 95% B, 2.0 min; 60% B, 3.0 min; 35% B, 5 min; hold 0.25 min; 0% B, 6 min; hold 0.5 min; 100% B, 7.8 min. The mass analyzer parameters included: Drying gas temperature, 250 °C; sheath gas temperature, 325 °C; nebulizer, 45 psi; skimmer, 50 V; octopole radio frequency, 750 V; scan rate, 5 spectra/s. For the ESI positive mode experiment, MS spectra were acquired over a voltage gradient of capillary 3500 V, nozzle 2000 V, and fragmentor 100 V. For ESI negative mode experiment, mass spectra were acquired over a voltage gradient of capillary 3000 V, nozzle 2000 V, and fragmentor 80 V. LC-MS data analysis: Prior to pre-processing each dataset, the extracted ion (XIC) and total ion (TIC) chromatograms for pooled quality control (QC) samples were examined to inspect consistency of retention time and ionization levels throughout. Following acquisition, mass feature bins were defined by targeted selection using a compound reference library which allowed partitioning of the *m*/*z* vs. retention time (RT) matrices into fixed width using Agilent Masshunter Profinder B.08.00 (Agilent Technologies, Wilmington, DE, USA). Bins were manually inspected to confirm consistent, reproducible integration for each compound of interest across all samples. Targeted ion selection and alignment parameters for logical binning of the input data were restricted to ion mass range ± 5.0 mDa and retention time ± 0.4 min. Following pre-processing, the ion abundance for each sample was corrected to sample-specific weight for tissue. Values were corrected to sample mass and internal standard response, then normalized to the median, and log-transformed to perform Welch corrected *t*-test. Vista Flux software Version 1.0 (Agilent Technologies, Wilmington, DE, USA) was used to conduct ^13^C-isotopologue and natural abundance comparative analysis for labeled-substrate experiments.

### 4.5. Hyperpolarized Magnetic Resonance Spectroscopy Imaging

The hyperpolarized MRSI was performed as previously described [22]. Samples of [1-^13^C] pyruvic acid (30 µL) containing 15 mM of OX063 and 2.5 mM of the gadolinium chelate ProHance (Bracco Diagnostics, Milano, Italy) were polarized in the hypersense DNP polarizer (Oxford Instruments, Abingdon, UK). After 40–60 min, the hyperpolarized sample was rapidly dissolved in 4.5 mL of a superheated Tris-based alkaline buffer. NaOH was added to the dissolution buffer to be pH 7.4 after mixture with [1-^13^C] pyruvic acid. Hyperpolarized [1-^13^C] pyruvate solution (96 mM) was intravenously injected through a catheter placed in the tail vein of mouse (12 µL/g body weight). The mice were anesthetized with isoflurane (4% for induction and 1.5–2.5% for maintaining anesthesia) in medical air and were positioned prone with their tumor-bearing head placed inside the resonator. During MRI measurements, the breathing rate of the mouse was monitored with a pressure transducer (SA Instruments Inc., Stony Brook, NY, USA) and was maintained at 70 ± 20 breaths per minute. Core body temperature was also monitored with a nonmagnetic rectal temperature probe (FISO) and was maintained at 36 ± 1 °C with a flow of warm air. Hyperpolarized ^13^C MRI studies were performed on a 3T scanner (MR Solutions, Guildford, UK) using a home-built ^13^C quadrature coil placed inside of a linear coil for ^1^H. ^13^C two-dimensional spectroscopic images were acquired 30 s after the start of the pyruvate injection, with a 28 × 28 mm^2^ field of view in an 8 mm slice through the tumor, a matrix size of 14 × 14, spectral width of 3330 Hz, repetition time of 85 ms, and Gaussian excitation pulse with a flip angle of 10°.

### 4.6. Magentic Resonance Spectroscopy Imaging Data Analysis

^13^C chemical shift images and ^1^H anatomical images were merged using MATLAB software (version 9.2). Displayed representative spectra were derived from spectra in tumor regions. A heat-map of the [1-^13^C] pyruvate, [1-^13^C] lactate, and [1-^13^C] lactate to [1-^13^C] pyruvate ratio was calculated in each pixel of chemical shift images and the resolution was digitally enhanced form matrix size of 14 × 14 to 32 × 32. The median value of the lactate:pyruvate ratio was calculated from chemical shift images in the tumor region. All MRS data analysis except the merging process was performed using Image J software (version 1.51).

### 4.7. Western Blot

Protein extraction was performed using proteinase inhibitor-containing RIPA lysis buffer (Santa Cruz Biotechnology, Dallas, TX, USA). About 15 μg extracted protein was used for western blotting with anti-LDHA (Cell Signaling, Danvers, MA, USA) #2012, 1:1000) and anti-β-Actin antibody (Sigma, St. Louis, MO, USA) #A5441, 1:5,000) as internal control. Western blotting results were detected by enhanced chemiluminescence substrate (46,640, Thermo Fisher) and imaged by BioRad ChemiDoc Imager (BioRad Laboratories, Inc., Hercules, CA, USA). Band intensities were analyzed by Image J (Version 1.51w).

### 4.8. Statistical Analysis

Correlation maps, PCA, and significance of statistical tests was assessed using Prism GraphPad (version 7) and in-house R scripts. Heat maps and correlation maps were generated using MetaboAnalyst version 4.0 [44] and the *corrplot* R package (https://cran.r-project.org/web/packages/corrplot/), respectively.

## 5. Conclusions

IDH1 mutant tumors display a distinct metabolic profile from normal tissue, besides the presence of 2HG. Indeed, several amino acids were dysregulated in the malignant region. However, these tumors have an active glycolytic pathway similar to those encountered in the normal mouse brain. Interestingly, the IDH1*^mut^* mouse model investigated herein shows an enhanced flux of glutamine towards lactate involving the synthesis of glucose, revealing a major rewiring of its metabolism.

## Supplementary Materials

The following are available online at https://www.mdpi.com/2072-6694/12/6/1633/s1, Figure S1: Quantification of metabolites directly involved with glutamate in the contralateral and tumor regions of the GEMM employed herein. Metabolic levels were computed through integration of specific regions of ^1^H NMR spectra, and the assessment of significance difference between regions was conducted using a *t*-test followed by Welch correction; ***, *p* < 0.001 (data displayed as mean ± SD, *n* = 5–6 mice). Figure S2: Glycolytic activity in the GEEMs: (A) and (B) MRI of the mouse brain with overlaid ^13^C NMR spectra for each voxel and the averaged spectrum for the tumor region. (C) Glucose levels computed by LC-MS for those regions (data displayed as mean ± SD, *n* = 3–6 mice). Figure S3: Molecular description and performance of the GEMM. (A) Immunoblotting of the transfected DF-1 cells confirms the successful expression of the transgenes. (B) Kaplan–Meier analysis shows the disease outcome of mouse glioma model. (C) Histology illustration of IDH1*^mut^* mouse glioma models. Figure S4: Lactate dehydrogenase expression in GEEM mice. (A) Western blot images for Figure 2D. Lanes 1–3: Contralateral region of 3 transfected mice; lanes 4–6: Tumor region of 3 transfected mice; line 7: right hemisphere from a control mouse and line 8: left hemisphere from a control mouse. (B) Lines as in (A) from two experiments.
