# Supplementary material for: Metabolic Landscape of a Genetically Engineered Mouse Model of IDH1 Mutant Glioma
Source: Cancers (Basel). 2020 Jun 19;12(6):1633. doi: 10.3390/cancers12061633 (PMC7352932; doi:10.3390/cancers12061633)
Supplement: Supplementary file 1 [file cancers-12-01633-s001.pdf]

cancers

MDPI

Article

Metabolic Landscape of a Genetically Engineered Mouse Model of IDH1 Mutant Glioma

Victor Ruiz-Rodado, Tomohiro Seki, Tyrone Dowdy, Adrian Lita, Meili Zhang, Sue Han, Chunzhang Yang, Murali K. Cherukuri, Mark R. Gilbert and Mioara Larion

Supplementary Material

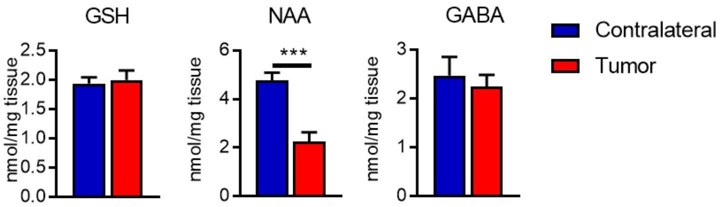

Figure S1: Quantification of metabolites directly involved with glutamate in the contralateral and tumor regions of the GEMM employed herein. Metabolic levels were computed through integration of specific regions of ^{1}H NMR spectra and the assessment of significance difference between regions was conducted using a t-test followed by Welch correction; ***, $p < 0.001$ (data displayed as mean ± SD, n = 5–6 mice).

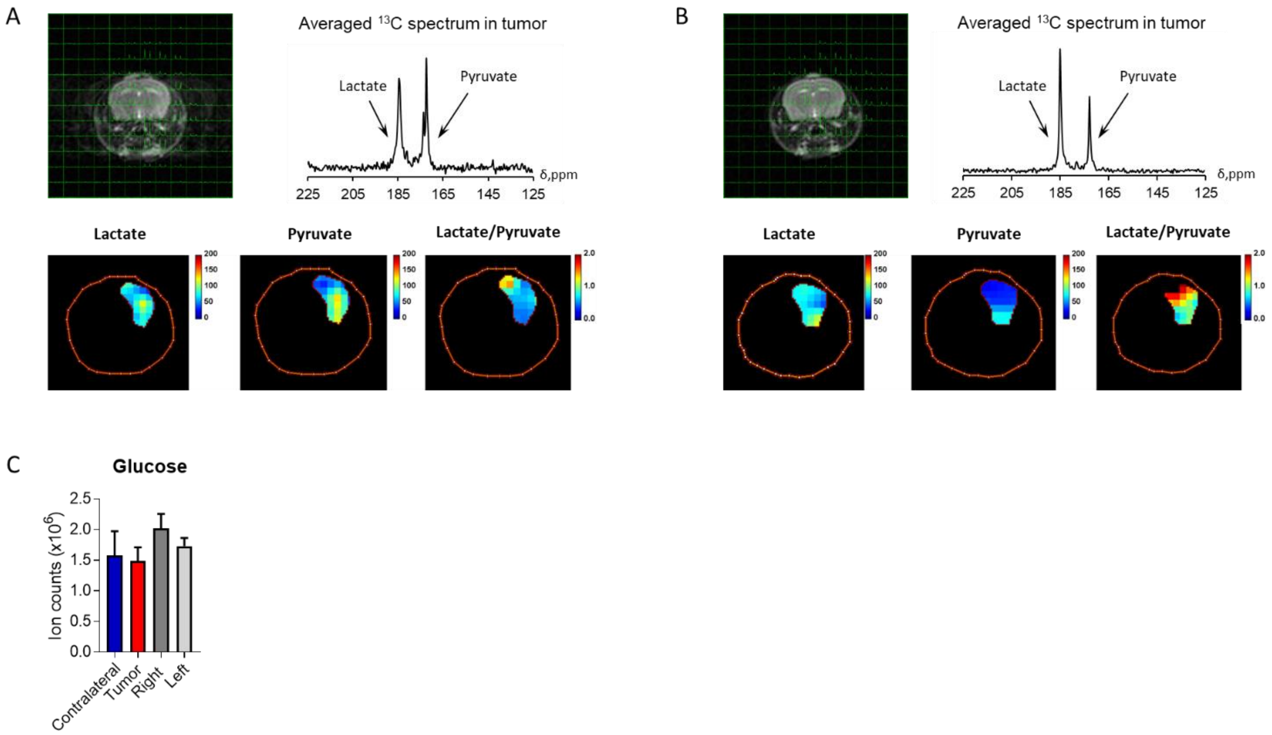

Figure S2: Glycolytic activity in the GEEMs: (**A**) and (**B**) MRI of the mouse brain with overlaid ^{13}C NMR spectra for each voxel and the averaged spectrum for the tumor region. (**C**) Glucose levels computed by LC-MS for those regions (data displayed as mean ± SD, n = 3–6 mice).

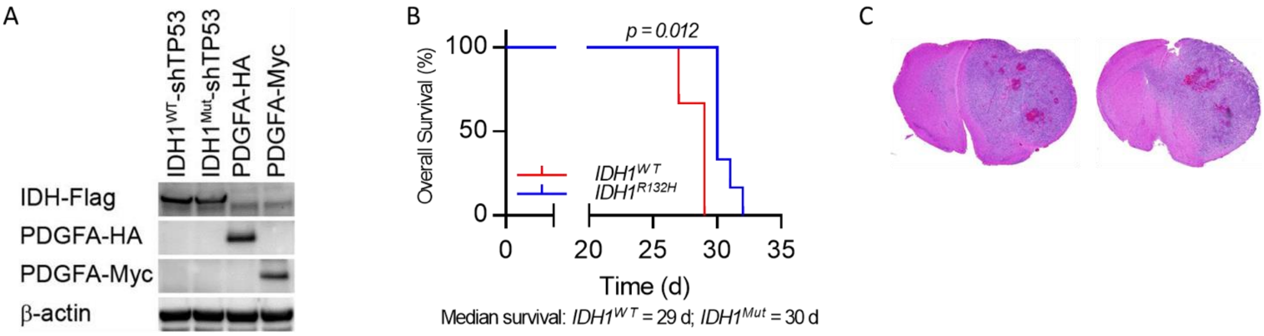

Figure S3: Molecular description and performance of the GEMM. **(A)** Immunoblotting of the transfected DF-1 cells confirms the successful expression of the transgenes. **(B**) Kaplan-Meier analysis shows the disease outcome of mouse glioma model. (**C**) Histology illustration of IDH1^{mut} mouse glioma models.

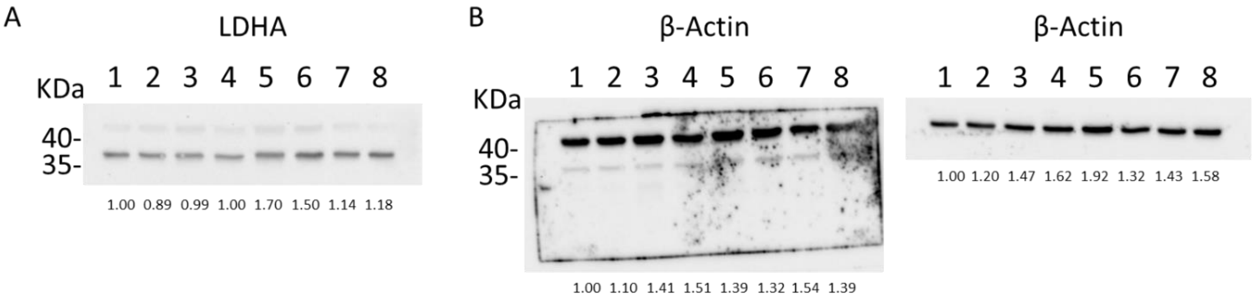

Figure S4: Lactate dehydrogenase expression in GEEM mice. (**A**) Western blot images for Figure 2D. Lanes 1–3: Contralateral region of 3 transfected mice; lanes 4–6: Tumor region of 3 transfected mice; line 7: right hemisphere from a control mouse and line 8: left hemisphere from a control mouse. (**B**) Lines as in (**A**) from two experiments.
